# Computational Models Used to Predict Cardiovascular Complications in Chronic Kidney Disease Patients: A Systematic Review

**DOI:** 10.3390/medicina57060538

**Published:** 2021-05-27

**Authors:** Alexandru Burlacu, Adrian Iftene, Iolanda Valentina Popa, Radu Crisan-Dabija, Crischentian Brinza, Adrian Covic

**Affiliations:** 1 Institute of Cardiovascular Diseases Prof. Dr. George I.M. Georgescu, 700503 Iasi, Romania; alexandru.burlacu@umfiasi.ro (A.B.); crischentian-branza@email.umfiasi.ro (C.B.); 2 Faculty of Medicine, University of Medicine and Pharmacy Grigore T Popa, 700115 Iasi, Romania; radu.dabija@umfiasi.ro (R.C.-D.); adrian.covic@umfiasi.ro (A.C.); 3 Romanian Academy of Medical Sciences, 030167 Bucharest, Romania; 4 Faculty of Computer Science, Alexandru Ioan Cuza University, 700259 Iasi, Romania; adiftene@info.uaic.ro; 5 Pulmonology Department, Clinic of Pulmonary Diseases, 700115 Iasi, Romania; 6 Nephrology Clinic, Dialysis, and Renal Transplant Center, ‘C.I. Parhon’ University Hospital, 700503 Iasi, Romania

**Keywords:** chronic kidney disease, artificial intelligence, cardiovascular complications, prevention, predictive models

## Abstract

*Background and objectives:* cardiovascular complications (CVC) are the leading cause of death in patients with chronic kidney disease (CKD). Standard cardiovascular disease risk prediction models used in the general population are not validated in patients with CKD. We aim to systematically review the up-to-date literature on reported outcomes of computational methods such as artificial intelligence (AI) or regression-based models to predict CVC in CKD patients. *Materials and methods:* the electronic databases of MEDLINE/PubMed, EMBASE, and ScienceDirect were systematically searched. The risk of bias and reporting quality for each study were assessed against transparent reporting of a multivariable prediction model for individual prognosis or diagnosis (TRIPOD) and the prediction model risk of bias assessment tool (PROBAST). *Results:* sixteen papers were included in the present systematic review: 15 non-randomized studies and 1 ongoing clinical trial. Twelve studies were found to perform AI or regression-based predictions of CVC in CKD, either through single or composite endpoints. Four studies have come up with computational solutions for other CV-related predictions in the CKD population. *Conclusions:* the identified studies represent palpable trends in areas of clinical promise with an encouraging present-day performance. However, there is a clear need for more extensive application of rigorous methodologies. Following the future prospective, randomized clinical trials, and thorough external validations, computational solutions will fill the gap in cardiovascular predictive tools for chronic kidney disease.

## 1. Introduction

Cardiovascular complications (CVC) are the leading cause of death in patients with chronic kidney disease (CKD) [1]. Individuals with CKD are most likely to die from CVC, regardless of the degree of renal failure [2]. Moreover, CKD appears to be a risk factor for CVC since kidney disease accelerates CVC development through uremic and non-uremic mechanisms [3]. In turn, CVC can contribute to renal failure progression, constituting an authentic vicious cycle [4,5]. Preventing CVC in CKD patients could significantly reduce mortality and delay disease progression by breaking this vicious circle [6].

However, common cardiovascular disease (CVD) risk prediction models used in the general population are not validated in patients with CKD, manifesting a low accuracy [1]. The latest cardiovascular (CV) risk management recommendations in CKD merely tackle blood pressure and lipid control, and limited data exist on interventions targeting other cardiovascular risk factors in CKD or explicit preventive strategies [7,8]. Thus, developing more robust strategies for preventing CVC development in CKD is much needed and would constitute a significant breakthrough. When “traditional” prevention measures reach their climax, the door to computational solutions such as artificial intelligence (AI) and regression-based methods seems to open [9].

Computational models [10] may substantially contribute to screening strategies for the prevention and early diagnosis of CVC in CKD. A computer-based model that could predict the imminent risk of myocardial infarction (MI) in a dialysis patient would significantly change the management strategies. Although numerous studies already researched AI/ML methods to predict different CVD types in the general population [11,12,13,14,15], fewer studies focused on developing CVC predictive models in CKD. General models may not suit the CKD clinical setting due to different CVC mechanisms involving a wide array of nontraditional risk factors, functional and structural alterations unique to CKD [16,17]. Subsequently, the data used to train prediction models for the general population might not fit CKD patients’ data.

No systematic review of computational models for CVC prediction in CKD has yet been carried out to our knowledge.

Therefore, our goals are (a) to conduct the first systematic review of studies that develop and assess computational models for the prediction of cardiovascular complications in CKD patients, (b) to evaluate the reporting quality and risk of bias of these studies for their potential to be integrated into clinical practice and guidelines, and (c) to stimulate and propose further research directions on computational algorithms for the early identification of complications. Our approach draws attention to the currently neglected clinical area of CVC in CKD and its burden on global healthcare and highlights how computational techniques could substantially ease burden through screening, prevention, and early diagnosis.

## 2. Materials and Methods

This study was conducted according to the PRISMA (Preferred Reporting Items for Systematic Review and Meta-Analysis) checklist [18].

### 2.1. Data Sources

The electronic databases of MEDLINE/PubMed, EMBASE, and ScienceDirect were systematically searched for relevant articles from inception until January 2021. The search query was (“Artificial intelligence” or “Machine learning” or “ML” or “AI”) and (“Chronic kidney disease” or “CKD” or “Renal insufficiency” or “ESRD” or “End-stage renal disease” or “Dialysis”) and (“Heart failure” or “Cardiac insufficiency” or “Coronary Artery Disease” or “CAD” or “Coronary syndrome” or “Coronary” or “Stable angina” or “Angina pectoris” or “Ischemic heart disease” or “IHD” or “Ischemic” or “Ischemia” or “Myocardial infarction” or “Infarction” or “Atrial fibrillation” or “AF” or “Stroke” or “Arrhythmia” or “Heart rate” or “Pulse” or “Sudden death” or “Sudden cardiac death” or “MACE” or “Cardiovascular prevention”).

### 2.2. Study Selection

The study selection process included identifying articles, removing duplicates, screening titles and abstracts, and assessing the selected full texts’ eligibility. Additionally, the reference lists of valuable articles were checked for studies of relevance. Original research articles were included if they evaluated computational methods such as AI/ML (random forests (RF), decision trees (DT), support vector machines (SVM), neural networks, K-nearest neighbor, and any other ML) or regression-based models to predict cardiovascular complications in CKD patients. Papers were eligible for inclusion if they provided predictive models for major adverse cardiovascular events (MACE), sudden cardiac death (SCD), ischemic heart disease (IHD), heart failure (HF), or arrhythmias in patients with CKD at any stage. Journal articles published with full text or abstracts in English were eligible for inclusion.

### 2.3. Data Extraction

The following data were extracted from the included studies: the population size and characteristics, the clinical setting, the algorithm, the number of predictors, the algorithms performance metrics, and the identified CV risks. When available, the study results or performance metrics were presented as hazard ratio (HR), c-statistic/Area Under the Receiver Operating Characteristics (AUC), confidence intervals (CIs), accuracy (ACC), sensitivity (SE), specificity (SP), or F1 score. Two authors extracted the data from study reports independently and in duplicate for each eligible study, with disagreements resolved by consensus.

### 2.4. Outcomes

The primary outcomes of interest were the AI/ML and regression-based predictions of CVC in CKD either through single or composite endpoints (MACE, SCD, IHD, HF, and arrhythmias). The secondary outcomes of interest included other CV-related predictions in the CKD population for which computational models have been described.

### 2.5. Quality Assessment

We assessed the reporting quality of non-randomized studies against the TRIPOD (transparent reporting of a multivariable prediction model for individual prognosis or diagnosis) statement [19]. We assessed the risk of bias for non-randomized studies by applying PROBAST (prediction model risk of bias assessment tool) [20].

## 3. Results

**Figure 1:**
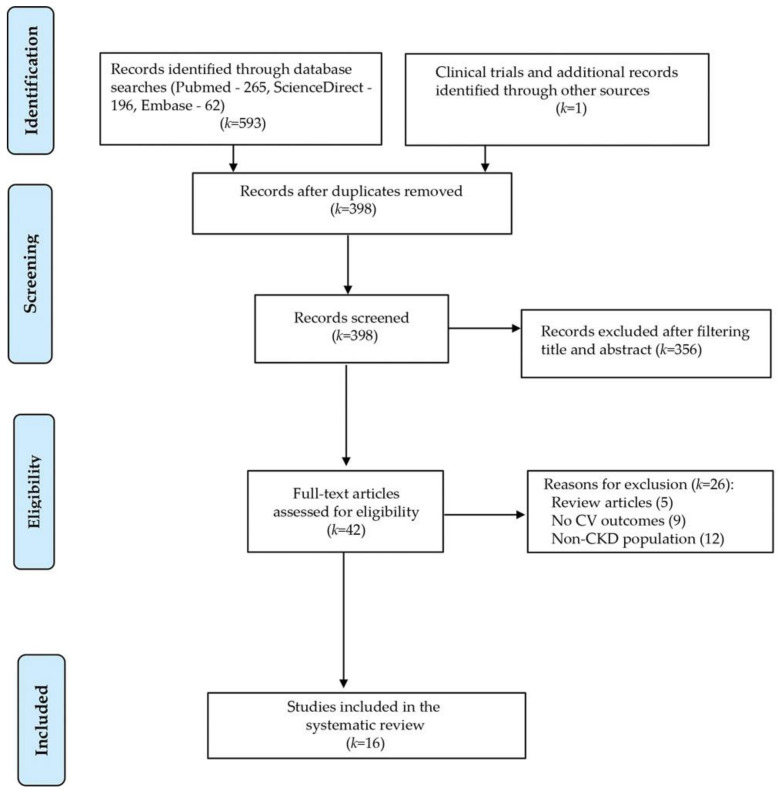
Study selection process and number of papers included in the systematic review.

Our electronic search, which was last updated on 12 February 12, 2021, retrieved 524 records (MEDLINE/Pubmed (265), ScienceDirect (196), Embase (62), and other sources (1 ongoing clinical trial)). Of the 524 study records, 198 duplicates were removed. Of the resulting 398 studies, 356 were excluded based on title and abstract screening due to the subject’s irrelevance. We assessed 42 full-text articles. After removing review articles or studies that did not meet the inclusion criteria (no CV outcomes or non-CKD population), 15 non-randomized studies and one ongoing clinical trial were included. The study selection process and the number of papers identified in each phase are illustrated in a flowchart (Figure 1).

Table 1 summarizes the characteristics of the 16 included papers. All studies are retrospective [21]. Eleven studies are single-center. Of the 11 single-center studies, six papers use datasets with patients from the US [21,22,23,24,25,26], three papers include individuals from Spain [27,28,29], one article includes patients from Korea [30], and one paper includes patients from Portugal [31]. Three of the eleven single-center studies [25,28,29] proposed ML and regression models based on the CRIC (Chronic Renal Insufficiency Cohort Study) [32] or NEFRONA [33] study cohorts. CRIC and NEFRONA studies aimed to examine risk factors for CKD progression and CVD among patients with established CKD and to evaluate novel biomarkers influencing cardiovascular events and mortality in all forms of CKD. The remaining five studies were multicenter. The research by De Gonzalo-Calvo et al. [34] was based on a dataset gathering patients from 300 centers in Europe, Canada, Australia, Brazil, Mexico, and South Korea. Matsushita et al. [35] validated the proposed models on the GCKD (German Chronic Kidney Disease) [36] and Hong Kong CKD cohorts [37]. The datasets used by Titapiccolo et al. [38] included patients from clinics located in Portugal and Spain. Goldstein et al. [39] collected patients’ dialysis data from the multicenter DaVita Inc. datasets (including 11 countries: US, Brazil, China, Colombia, Germany, Malaysia, Poland, Portugal, Saudi Arabia, Singapore, and the United Kingdom). Finally, the research by Mezzatesta et al. [40] was based on US and Italian datasets.

Regarding our systematic review’s primary outcomes of interest, 12 studies were found to perform AI/ML and regression-based CVC predictions in CKD either through single or composite endpoints.

Five studies proposed AI/ML models to predict CVC in CKD through composite endpoints. Various compounded endpoints such as MACE (major adverse cardiovascular events) in postoperative end-stage renal disease (ESRD) patients [30], or combinations of CV events (CV death, nonfatal MI, nonfatal stroke, and CV hospitalizations) in hemodialysis [34,38] or stage 3–5 CKD [35] patients have been predicted using AI/ML tools. Fernandez-Lozano et al. [27] proposed a model to predict overall CVC as the leading cause of morbidity and mortality in CKD patients.

Of the included studies, seven papers developed AI/ML models to predict single CV endpoints concerning the most frequent and vital CVC in CKD (SCD, IHD, HF, and arrhythmias). The proposed models predict SCD in hemodialysis patients [39,40], IHD, HF, or arrhythmias in hemodialysis patients [40]; prognostic proteins associated with HF in CKD [21]; death risk in ESRD patients with congestive HF [22]; HF admissions in individuals with CKD [23]; the safety and efficiency of low-dose angiotensin-converting-enzyme inhibitors (ACEIs) and angiotensin II receptor blockers (ARBs) in CKD patients with HF and reduced ejection fraction [24]; and incident atrial fibrillation (AF) in CKD patients without prior AF [25].

Four studies were found to put forward AI/ML solutions for other CV-related predictions in the CKD population regarding our secondary outcomes of interest. As such, models have been proposed for the prediction of atheromatous CVC in stage 3–5 CKD patients [28], the discrimination between proatherogenic lipid profile in CKD vs. controls [29], the stroke risk in continuous ambulatory peritoneal dialysis (CAPD) patients [31], and hyperkalemia detection from the ECG in stage 3–5 CKD patients [26].

Overall, the included studies reported the models’ performances using the area under the receiver operator characteristic curve (AUC). All reported AUCs are more generous than 0.71. Few studies reported ACC, SE, SP, or F1 scores as the performance metrics. The reported ACCs ranged from 64% to 99.88%.

The most used algorithm by the included studies was the RF. DT, SVM, classification and regression trees (CART), and deep convolutional neural network (DCNN) are other methods used to implement the AI/ML solutions in the included studies.

Precise data on population, outcomes, algorithms, and model performance are illustrated in Table 1.

The adherence to reporting standards was moderate (<50% adherence in half of the studies) for 20 of 29 TRIPOD items (see Table S1). Overall, the publications adhered to between 15% and 85% of the TRIPOD items, with a mean of 51%.

The overall risk of bias assessed using PROBAST led to 68% of studies classified as high risk or unclear risk of bias (Table S2).

## 4. Discussion

This is the first study that adds a systematic review of computational solutions to predict CVC outcomes in the CKD population to the literature. Our attempt aims to fill the gap in specific systematized predictive tools for CVC in CKD. After surveying the directions and assessing the quality of the included studies, we provide a balanced perspective on the present-day status and the future of computational predictive methods in the CKD clinical setting.

**Figure 2:**
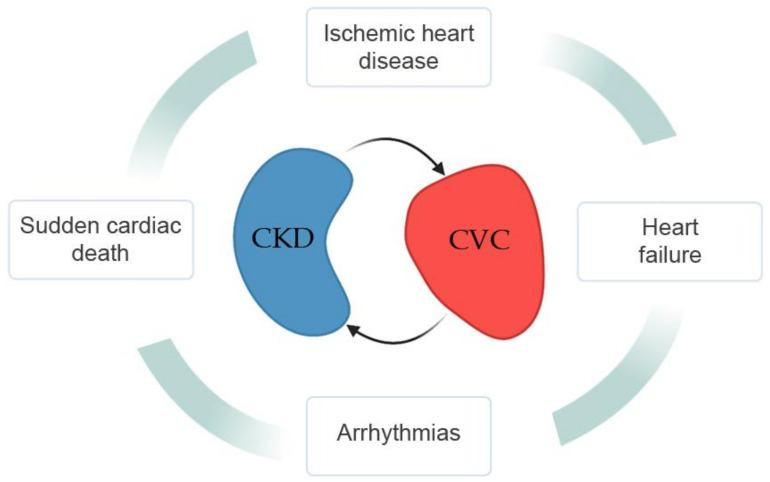
The four main manifestations of type-4 cardiorenal syndrome.

Type-4 cardiorenal syndrome refers to CV involvement in CKD. After a thorough literature survey, we identified four main manifestations of type-4 cardiorenal syndrome: sudden cardiac death, arrhythmias, ischemic heart disease, and heart failure [41,42] (Figure 2).

First, AI/ML and regression-based solutions dealing with SCD prediction in CKD follow two directions: short-term predictions of SCD one day after a hemodialysis session [39] and long-term predictions in hemodialysis patients (2.5-year follow-up) [40]. Goldstein et al. [39] and Mezzatesta et al. [40] adhered to 70% and 40% of the TRIPOD criteria, respectively. Unlike Goldstein et al. [39], Mezzatesta et al. [40] did not clearly describe the eligibility criteria for participants or the transparent handling of predictors in the analysis. However, Goldstein et al. [39] did not use external data sets to validate their models, while Mezzatesta et al. [40] tested their models on two different datasets (Italian and American). CKD patients presented higher mortality rates due to SCD [43], with major cardiac events representing 50% of death causes in CKD patients [44]. After further improvement and rigorous validations, these predictive tools will make it possible to ameliorate mortality rates due to CV events in CKD patients by helping to deal with serious adverse events, such as SCD, before they occur.

Second, automated learning models for arrhythmia predictions in CKD are oriented toward predicting incident AF (7.3-year follow-up) in CKD patients without prior AF [25] and unspecified arrhythmias (2.5-year follow-up) in hemodialysis patients [40]. Similar to Mezzatesta et al. [40], Zelnick et al. [25] did not specify how predictors are handled in their analysis. Unlike Mezzatesta et al. [40], Zelnick et al. [25] did not describe how missing data and the validation sets were managed. However, Zelnick et al. [25] was a conference abstract, and in all likelihood, some of the information about the models may not be included in this type of communication. Both studies have similar adherences to TRIPOD items (40–45%). Arrhythmia was reported to be as prevalent as 78% in CKD patients [45]. While a percentage of arrhythmias are at the root of sudden death, benign arrhythmias such as AF have gained more and more attention due to their high prevalence in CKD patients and the complex interrelation between stroke and hemorrhagic risks and due to opposing therapeutic strategies. One study reported that the 1-year mortality in dialysis patients with AF is twice as high as those without AF [46]. Thus, such models oriented toward predicting the risks of arrhythmia (and particularly AF) in CKD patients may consistently reduce these unwanted consequences.

Third, one AI/ML model to assess the risk of IHD in CKD is directed towards predicting long-term IHD (2.5-year follow-up) in hemodialysis patients [40]. IHD is one of the most frequent CVCs in hemodialysis, as shown by the HEMO study [47]. The HEMO study demonstrated that most of the hospitalizations of hemodialysis patients were for acute coronary syndrome. Such predictions may lead to optimized preventive strategies against coronary events, better distribution of resources, and intensified monitoring of the patients identified as high risk.

Fourth, automated learning methods dealing with HF predictions in CKD explore the potential of AI solutions to predict not only the risk of incident HF [21,23,40] but also the outcomes of HF [22] and the effectiveness of standard therapies in the particular CKD setting [24]. Models to predict long-term risk of HF in hemodialysis patients using SVM [40] or HF admissions in patients with CKD based on remote IoT (Internet of Things) sensors [23] have been described. Additionally, to assess the risk of occurrence, a model identifies prognostic proteins associated with HF in CKD by random survival forest regression [21]. Regarding the prediction of outcomes in CKD patients diagnosed with HF, a model to predict all-cause mortality in ESRD patients with congestive HF has been proposed by Akbilgic et al. [22]. An ongoing clinical trial uses AI/ML-based methods to predict the safety and efficiency of low-dose ACEIs and ARBs in CKD patients with HF and reduced ejection fraction [24]. Akbilgic et al. [22] had a high adherence to TRIPOD criteria (75%), while Gowda et al. [23], Dubin et al. [21], and Ahmed et al. [24] had poor adherences, with less than 30% of the criteria met. Akbilgic et al. [22] used an impressive dataset of 14,800 patients, whereas Gowda et al. [23] included data from only 117 patients. The CRIC study [48] proved that ejection fractions significantly decline during more advanced stages of CKD. Moreover, the atherosclerosis risk in communities (ARIC) population study [49] showed that cardiac insufficiency starts earlier in the process as lower CKD stages are also associated with varying degrees of HF. Although AI/ML-based studies tackling HF predictions in CKD are of heterogeneous quality and bias, they pinpoint the various directions in which automated learning could impact the management of CKD patients with high HF risk starting from lower CKD stages.

Fifth, composite endpoint studies are the starting point for designing CV risk stratification models. Computational solutions [27,30,34,35,38] have also been described to predict composite CV endpoints, such as MACE, in CKD patients. Composite endpoint studies are also heterogeneous regarding reporting transparency and bias, with a TRIPOD adherence ranging from 20% to 70%. Jeong et al. [30] and Titapiccolo et al. [38] are the only studies out of the 16 included papers that report the issue of imbalanced data and describe how to handle it. Studies assessing composite models will shape global CV risk stratification policies, ultimately leading to treatment personalization and improved cardiovascular system preservation.

Finally, we found four studies that put forward computational solutions for other CV-related predictions in the CKD population. Hyperkalemia is common in CKD patients and is associated with fatal arrhythmias [26]. Therefore, Galloway et al. [26] proposed a deep convolutional neural network (DCNN) to detect hyperkalemia from the electrocardiogram (ECG) in stage 3–5 CKD patients. Additionally, Bermudez-Lopez et al. [29] described an RF model to discriminate between proatherogenic lipid profiles in CKD vs. controls. The model of Bermudez-Lopez et al. [29] outlines the particular proatherogenic lipid profile in CKD that constitutes the basis for identifying the most appropriate lipid-lowering therapy for the prevention of CVC in CKD (knowing that statins are not very useful in this setting).

The main limitations of the computational techniques used for the prediction of CVC in CKD are represented by moderate adherence to standard quality criteria and moderate transparency regarding essential steps of building automated learning models: lack of clarity on the issues of imbalanced data for 87.5% of the included studies, unclear definition and handling of predictors for 50% of the studies, vague eligibility criteria for 37.5%, and non-random or insufficient external validation). Moreover, different ascertainments and definitions of CVC, and non-rigorous clinical classifications of CV diseases in the included studies (lack of differentiation between acute versus chronic disease, specific types of arrhythmia, and myocardial infarction versus angina) could be a source of significant bias. Additionally, all papers considered in this systematic review were retrospective.

## 5. Conclusions

In this systematic review, we explored the potential of computer-based models in predicting CV risk and outcomes in CKD, and we identified palpable trends in areas of clinical promise. In particular, type-4 cardiorenal syndrome manifestations may be managed more efficiently using computational predictive tools to prevent sudden cardiac death, arrhythmias, ischemic heart disease, and heart failure in CKD. We pointed out the possible benefits of these modern methods on public health. We also identified a clear need for more extensive application of rigorous methodologies. The call for more thorough methodologies and the need for prospective, randomized clinical trials and thorough external validations clearly outline the future research directions in this area. Considering the present-day performances, the interest shown so far, and the future perspectives, we firmly believe that computational solutions will fill the gap in cardiovascular predictive tools for chronic kidney disease patients in the future.

## Figures and Tables

**Table 1:** Summary of the included studies.

Author, Year	Population	Outcomes	Sample Size/Predictors No.	Algorithm	Performance
Composite CV outcomes					
de Gonzalo-Calvo et al. 2020 [34]	Hemodialysis	Time to CV death, nonfatal MI, or nonfatal stroke (24 months follow-up)	778/8	DT using the CART algorithm	AUC: 0.71
Matsushita et al. 2020 [35]	Moderate CKD (GCKD cohort): 5-year follow-up	MI or fatal CHD or stroke	5217 (validation set)	CKD Patch (Linear regression + Statistical methods)	AUC: 0.698
	Stage 3–5 CKD (Hong Kong CKD): 10-year follow-up		300 (validation set)		AUC: 0.73
Titapiccolo et al. 2013 [38]	Incident hemodialysis	CV events (CV mortality, insurgence of new CV co-morbidity, or CV hospitalization) in the next six months	4246/39	RF	AUC: 0.737 ± 1.2; ACC: 67.3 ± 2.8%; SE: 69.2 ± 3.3%; SP: 67.3 ± 2.8%
Jeong et al. 2021 [30]	Postoperative ESRD patients	MACE (1 month postoperatively)	3220/40	RF	F1 score: 0.797
Fernandez-Lozano et al. 2018 [27]	Peritoneal dialysis	CVC prediction	114	Generalized Linear Model	AUC: 0.96
Sudden cardiac death (SCD)					
Goldstein et al. 2014 [39]	Hemodialysis	Sudden cardiac death the day of or day after a dialysis session	1796/72	RF	AUC: 0.799
Mezzatesta et al., 2019 [40]	Hemodialysis	CV death (2.5-year follow-up)	861/23	SVM + RBF kernel	ACC: 80%
Ischemic heart disease (IHD)					
Mezzatesta et al. 2019 [40]	Hemodialysis	IHD (2.5-year follow-up)	522/29	SVM + RBF kernel	ACC: 95.25%
			2677/23		ACC: 92.15%
Heart failure (HF)					
Dubin et al. 2018 [21]	CKD	Prognostic proteins associated with HF in CKD	364	RSF regression + Cox survival analysis	Angiopoietin-2: HR 1.45 [1.33, 1.59] Spondin-1: HR 1.13 [1.06, 1.20]
Mezzatesta et al. 2019 [40]	Hemodialysis	HF (2.5-year follow-up)	522/29	SVM + RBF kernel	ACC: 93%
			2677/23		ACC: 64%
Akbilgic et al. 2019 [22]	ESRD patients with congestive HF	30-, 90-, 180-, and 365-day all-cause mortality	14800/49	RF	AUC: 0.683, 0.716, 0.725, and 0.725 (risk of death within the 4 different time windows)
Gowda et al. 2020 [23]	CKD	HF admissions in patients with CKD (1-year follow-up)	117	Remote IoT sensors	Significant decrease in HF admissions after implantation
Ahmed et al. [24]	CKD patients with HF and reduced ejection fraction	Safety and efficiency prediction of low-dose ACEIs and ARBs	Not available	ML algorithm (unspecified)	Not available (study ongoing)
Arrhythmias					
Zelnick et al. 2020 [25]	CKD patients without prior AF	Incident AF	2690/32	Lasso regression	AUC: 0.76
Mezzatesta et al. 2019 [40]	Hemodialysis	Arrhythmia (2.5-year follow-up)	522/29	SVM + RBF kernel	ACC: 95%
			2677/23		ACC: 67%
Other CV-related predictions					
Forné et al. 2020 [28]	Stage 3–5 CKD	Atheromatous CVC (4-year follow-up)	1366/38	RSF	AUC: 0.744
Bermudez-Lopez et al. 2019 [29]	Stage 3–5 CKD + Controls	Discriminate between proatherogenic lipid profile in CKD vs. controls	395/10	RF	AUC: 0.789
Rodrigues et al. 2017 [31]	CAPD	Stroke risk	850/7	K-nearest neighbor	ACC: 99.65%; SE: 95.35%; SP: 99.88%
Galloway et al. 2019 [26]	Stage 3–5 CKD	Hyperkalemia detection from the ECG	61,965 ECG-potassium pairs (validation set)	DCNN	AUC: 0.853–0.883

Cardiovascular (CV); Myocardial infarction (MI); Decision tree (DT); Classification and Regression Tree (CART); Area under the receiver operating characteristic curve (AUC); Chronic kidney disease (CKD); German Chronic Kidney Disease (GCKD); Coronary heart disease (CHD); Random forest (RF); Accuracy (ACC); Sensitivity (SE); Specificity (SP); End-stage renal disease (ESRD); Major adverse cardiovascular events (MACE); Cardiovascular complications (CVC); Support vector machine (SVM); Radial basis function (RBF); Systolic blood pressure (SBP); Ischemic heart disease (IHD); Heart failure (HF); Random survival forest (RSF); Hazard ratio (HR); Internet of Things (IoT); Angiotensin-converting-enzyme inhibitors (ACEIs); Angiotensin II receptor blockers (ARBs); Atrial fibrillation (AF); Continuous Ambulatory Peritoneal Dialysis (CAPD); Electrocardiogram (ECG); Deep convolutional neural network (DCNN).

## Supplementary Materials

The following are available online at https://www.mdpi.com/article/10.3390/medicina57060538/s1, Table S1: Quality assessment data for each study according to TRIPOD criteria. Table S2: The risk of bias assessment for each included study based on PROBAST criteria.
