# Supplementary material for: Computational Models Used to Predict Cardiovascular Complications in Chronic Kidney Disease Patients: A Systematic Review
Source: Medicina (Kaunas). 2021 May 27;57(6):538. doi: 10.3390/medicina57060538 (PMC8227302; doi:10.3390/medicina57060538)
Supplement: Supplementary file 1 [file medicina-57-00538-s001.zip › medicina-1210352-supplementary.pdf]

Table S1. Quality assessment data for each study according to TRIPOD criteria.

	**Methods**													**Results**							**Total**
	**Source of Data**		**Participants**		**Outcome**		**Predictors**		**Sample Size**	**Missing Data**	**Analysis methods**			**Participants**		**Model development**		**Model specification**		**Model performance**	
Reference (included studies)	4a	4b	5a	5b	6a	6b	7a	7b	8	9	10a	10b	10d	13a	13b	14a	14b	15a	15b	16	
de Gonzalo-Calvo et al.	Yes	No	Yes	Yes	Yes	Yes	Yes	No	Yes	Yes	Yes	Yes	No	Yes	Yes	Yes	No	No	No	Yes	70%
Matsushita et al.	Yes	No	Yes	Yes	Yes	No	No	No	Yes	No	No	Yes	No	Yes	No	No	No	No	No	Yes	40%
Titapiccolo et al.	Yes	Yes	Yes	Yes	Yes	No	Yes	No	No	Yes	Yes	Yes	Yes	Yes	No	Yes	No	No	No	Yes	65%
Jeong et al.	Yes	Yes	Yes	Yes	No	No	Yes	No	Yes	No	Yes	Yes	Yes	No	No	Yes	No	No	No	Yes	55%
Fernandez-Lozano et al.	Yes	No	Yes	No	No	No	No	No	No	No	No	Yes	No	No	No	Yes	No	No	No	No	20%
Goldstein et al.	Yes	Yes	Yes	Yes	Yes	No	Yes	No	Yes	Yes	Yes	Yes	Yes	Yes	Yes	Yes	No	No	No	No	70%
Mezzatesta et al.	Yes	No	Yes	No	Yes	No	No	No	Yes	Yes	No	Yes	Yes	No	No	Yes	No	No	No	No	40%
Akbilgic et al.	Yes	Yes	Yes	Yes	Yes	No	Yes	No	Yes	Yes	Yes	Yes	Yes	Yes	Yes	Yes	No	No	No	Yes	75%
Dubin et al.	Yes	No	No	No	No	No	No	No	No	No	No	Yes	Yes	No	No	No	No	No	No	Yes	20%
Gowda et al.	Yes	No	Yes	No	Yes	No	No	No	No	No	No	Yes	Yes	No	No	Yes	No	No	No	No	30%
Ahmed et al.	Yes	Yes	No	No	Yes	No	No	No	No	No	No	No	No	No	No	No	NA	No	No	No	15%
Zelnick et al.	Yes	No	Yes	Yes	Yes	No	No	No	Yes	No	No	Yes	Yes	No	No	Yes	NA	No	No	Yes	45%
Forné et al.	Yes	Yes	Yes	Yes	Yes	No	Yes	Yes	Yes	Yes	Yes	Yes	Yes	Yes	Yes	Yes	Yes	No	No	Yes	85%
Bermudez-Lopez et al.	Yes	Yes	Yes	Yes	Yes	No	Yes	No	Yes	No	Yes	Yes	Yes	Yes	Yes	Yes	No	No	No	Yes	70%
Rodrigues et al.	Yes	Yes	Yes	No	No	No	Yes	No	Yes	No	Yes	Yes	Yes	No	No	Yes	No	No	No	Yes	50%
Galloway et al.	Yes	Yes	Yes	Yes	Yes	No	No	No	Yes	No	No	Yes	Yes	Yes	Yes	Yes	No	No	No	Yes	60%

Table S2. The risk of bias assessment for each included study based on PROBAST criteria.

Study	ROB*				Applicability (AP)			Overall	
	PRT**	PRD***	Outcome	Analysis	PRT	PRD	Outcome	ROB	AP
de Gonzalo-Calvo et al.	+	+	+	+	+	+	+	+	+
Matsushita et al.	+	-	+	-	+	+	+	-	+
Titapiccolo et al.	+	+	+	+	+	+	+	+	+
Jeong et al.	+	+	-	-	+	+	+	-	+
Fernandez-Lozano et al.	-	-	-	-	+	+	+	-	+
Goldstein et al.	+	+	+	+	+	+	+	+	+
Mezzatesta et al.	-	-	+	+	+	+	+	-	+
Akbilgic et al.	+	+	+	+	+	+	+	+	+
Dubin et al.	-	-	-	-	+	+	+	-	+
Gowda et al.	?	?	+	?	+	+	+	?	+
Ahmed et al.	?	?	+	?	+	+	+	?	+
Zelnick et al.	+	?	+	?	+	+	+	?	+
Forné et al.	+	+	+	+	+	+	+	+	+
Bermudez-Lopez et al.	+	+	+	-	+	+	+	-	+
Rodrigues et al.	-	+	-	-	+	+	+	-	+
Galloway et al.	+	-	+	-	+	+	+	-	+

ROB* = risk of bias; PRT** = participants; PRD*** = predictors;
+ indicates low ROB/low concern regarding applicability; - indicates high ROB/concern regarding applicability; ? indicates unclear ROB/unclear concern regarding applicability
